# Addiction to protein kinase Cɩ due to *PRKCI* gene amplification can be exploited for an aptamer-based targeted therapy in ovarian cancer

**DOI:** 10.1038/s41392-020-0197-8

**Published:** 2020-08-21

**Authors:** Hina Rehmani, Yue Li, Tao Li, Ravi Padia, Ozlem Calbay, Lingtao Jin, Huijun Chen, Shuang Huang

**Affiliations:** 1 Department of Biochemistry and Cancer Biology, Medical College of Georgia, Augusta University, Augusta, GA 30912 USA; 2 Department of Anatomy and Cell Biology, University of Florida College of Medicine, Gainesville, FL 32610 USA; 3 Department of Gynecology and Obstetrics, Zhongnan Hospital of Wuhan University, 430071 Wuhan, Hubei China

**Keywords:** Gynaecological cancer, Oncogenes

## Abstract

*PRKCI*, the gene for protein kinase Cι (PKCι), is frequently amplified in ovarian cancer and recent studies have shown that PKCι participates in ovary tumorigenesis. However, it is unknown whether PKCι is differentially involved in the growth/survival between *PRKCI*-amplified and non-amplified ovarian cancer cells. In this study, we analyzed ovarian cancer patient dataset and revealed that *PRKCI* is the only PKC family member significantly amplified in ovarian cancer and *PRKCI* amplification is associated with higher PKCι expression. Using a panel of ovarian cancer cell lines, we found that abundance of PKCι is generally associated with *PRKCI* amplification. Interestingly, silencing PKCι led to apoptosis in *PRKCI*-amplified ovarian cancer cells but not in those without *PRKCI* amplification, thus indicating an oncogenic addiction to PKCɩ in *PRKCI*-amplified cells. Since small-molecule inhibitors characterized to selectively block atypical PKCs did not offer selectivity nor sensitivity in *PRKCI*-amplified ovarian cancer cells and were even cytotoxic to non-cancerous ovary surface or fallopian tube epithelial cells, we designed an EpCAM aptamer-PKCι siRNA chimera (EpCAM-siPKCι aptamer). EpCAM-siPKCι aptamer not only effectively induced apoptosis of *PRKCI*-amplified ovarian cancer cells but also greatly deterred intraperitoneal tumor development in xenograft mouse model. This study has demonstrated a precision medicine-based strategy to target a subset of ovarian cancer that contains *PRKCI* amplification and shown that the EpCAM aptamer-delivered PKCι siRNA may be used to suppress such tumors.

## Introduction

Ranked fifth in cancer death among women, ovarian cancer cure rates have remained low over the past two decades due to unsuccessful detection of early-stage disease, stagnant methodologies of treatment and high relapse rates. In stage IV invasive epithelial ovarian cancer where cancer metastasizes to the lungs, bone, and brain, women have a meager 17% relative 5-year survival rate. Relapses are frequently found in ovarian cancer, with ~70% of patients relapsing within the first two years of diagnosis, even after surgery and standard first-line chemotherapy with carboplatin/paclitaxel. Alarmingly, stage I or II patients still have a 20–25% relapse rate.^1^ Second-line chemotherapy, to date, has been very disappointing in all forms of ovarian cancer as the disease most often becomes chemo-resistant. Accounting for more deaths than any other cancer of the female reproductive system, it is paramount to strategically offer ovarian cancer patients with precision medicine-based targeted therapies to improve outcome.^2^

Ovarian cancer, especially high-grade serous ovarian cancer (HGSOC) contains a host of copy number aberrations (CNAs) that can lead to the silencing or amplification of tumor suppressor genes or oncogenes respectively.^3–5^ Clinically, gene amplifications have prognostic and diagnostic usefulness as they have been proven as indicators to gauge tumorigenic processes and/or drug resistance potential of cancer.^6^ Protein kinase Cι (PKCι) is an atypical member of PKC family and its gene *PRKCI* is frequently amplified in ovarian cancer.^7^ Because of its ability to induce loss of apical–basal polarity and increase cyclin E,^8^ PKCι has previously been indicated as a potential oncogene in ovarian cancer.^9^ This notion is supported by several recent studies that PKCι is shown to maintain tumor-initiating cell phenotype,^10^ regulate YAP1 activity,^11^ and promote immune suppression in ovarian cancer.^12^ However, it has not been investigated whether PKCι is differentially required between ovarian cancer with or without *PRKCI* amplification. In other word, it has not been asked whether *PRKCI*-amplified ovarian cancer is addicted to PKCι.

Aptamer is the terminology used to describe single-stranded DNA or RNA (ssDNA or ssRNA)　that display high affinity and specificity for a variety of targets.^13^ A major advantage of aptamers is that they exhibit neither intrinsic toxicity nor immunogenicity.^14,15^ Another advantage is that they can be quickly and inexpensively generated through automated oligonucleotide synthesizes without batch-to-batch variations.^16^ An example of aptamers is the 19-nucleotide ssRNA epithelial cell adhesion molecule (EpCAM) aptamer that possesses the similar binding affinity as EpCAM antibodies and is efficiently internalized through receptor-mediated endocytosis.^17^ Currently, aptamer-siRNA chimera (AsiC) has emerged as an effective strategy to mediate gene silencing in a cell-type-specific delivery application.^18^ For instance, gp120 RNA aptamer was shown to specifically deliver anti-HIV siRNA into HIV-1 infected cells and suppress HIV-1 replication in a humanized mouse model of HIV.^19^ Since EpCAM is overexpressed in more than 70% ovarian cancer and its expression in the peritoneal cavity appears to be tumor specific,^20,21^ we reason that EpCAM aptamer may be an effective and ideal vehicle to deliver therapeutic siRNAs to suppress ovary tumorigenecity.

The objectives of this study were to test the hypotheses that *PRKCI* amplification offers a unique opportunity to stratify patients for PKCι-targeted precision medicine therapy and AsiC represents a promising tool for such strategy. With the aid of both in vitro and in vivo experimental models, we showed that an EpCAM aptamer and PKCι siRNA chimera (EpCAM-siPKCι aptamer) not only induced apoptosis in *PRKCI*-amplified ovarian cancer cells but also suppressed intraperitoneal ovary tumor development. Our study may offer a promising precision medicine modality for a subset of ovarian cancer with *PRKCI* amplification.

## Results

### *PRKCI* amplification is unique and correlated with high PKCι expression in ovarian cancer

**Fig. 1:**
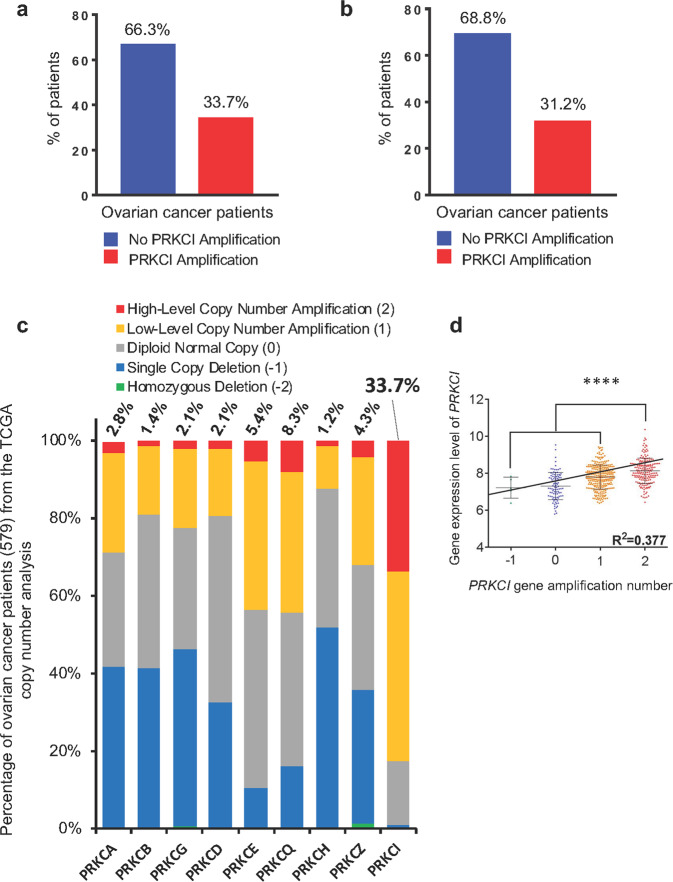
*PRKCI* amplification is unique and correlates with higher PKCι expression in ovarian cancer specimens. **a***PRKCI* amplification in ovarian cancer patients (total 579 ovarian serous cystadenocarcinoma patients included in TCGA dataset). **b***PRKCI* amplification in relapsed ovarian cancer patients (314 patients relapsed after receiving treatment in TCGA dataset). **c** Gene-level copy number estimation in ovarian cancer specimens was made using the GISTIC2 method using the TCGA dataset. The estimated values of −2, −1, 0, 1, and 2 represent homozygous deletion, single copy deletion, diploid normal copy, low-level copy number amplification, and high-level copy number amplification, respectively. **d** From 579 patients in the TCGA ovarian cancer dataset, 556 patients were found to have information for both *PRKCI* amplification status and PKCι expression. Data are means ± SD. Linear regression test was used to analyze correlation. ****P < 0.0001

Our investigation into the significance of PKCι in ovarian cancer began by analyzing datasets of serous cystadenocarcinoma ovarian cancer patients from the TCGA. We found that over 33% of total HGSOC patients and over 31% of relapsed patients harbored *PRKCI* amplification (Fig. 1a, b), indicating that the status of *PRKCI* amplification is not an outcome of cancer recurrence. Analyzing CNA of other members of PKC family revealed that *PRKCI* amplification is unique because no other member of the PKC family exhibits the level of amplification that *PRKCI* does (Fig. 1c). In fact, over 80% of patients have some form of *PRKCI* amplification (combined low-level and high-level), which is significantly higher than any other member of the PKC family (Fig. 1c). To determine the correlation between CNA of *PRKCI* and PKCι expression in HGSOC, we analyzed TCGA dataset to compare PKCι expression along the *PRKCI* copy number. Linear regression analysis showed that copy number of *PRKCI* was positively correlated with PKCι mRNA expression in HGSOC specimens (Fig. 1d). Considering unique amplification of *PRKCI*, it supports the notion that PKCι is specifically involved in ovary tumorigenesis.

**Fig. 2:**
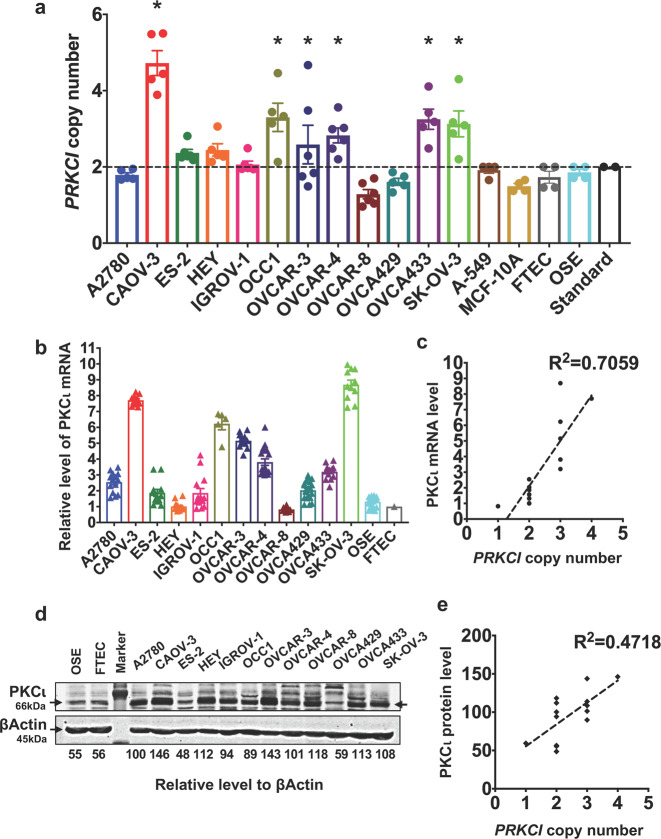
*PRKCI* amplification is associated with higher abundance of PKCι in established ovarian cancer cell lines. **a** Copy number analysis was performed to determine status of *PRKCI* amplification in ovarian cancer cell lines, immortalized ovarian cancer cell T29 (IOSE), OSE, FTEC, lung cancer A549 line, and immortalized breast epithelial MCF10A line. Copy number of each cell line was analyzed by CopyCaller software. Data are means ± SEM. Lines displaying statistically significant amplification for *PRKCI* are marked with *P < 0.05 versus Standard. The dotted line indicates the diploid status at copy number 2. Copy number analysis was performed with three technical replicates and at least two biological replicates. Error bars represent standard error of mean. **b** QRT-PCR was performed to measure the level of PKCι mRNA in ovarian cancer cell lines, OSE and FTEC cells. Level of βActin mRNA was used for standardization. Data are means ± SEM. **c** Linear regression was used to calculate the correlation between *PRKCI* amplification and PKCι mRNA in ovarian cancer cell lines. Level of PKCι mRNA was normalized to β-actin mRNA. **d** Cell lysates harvested from 12 ovarian cancer cell lines, OSE and FTEC cells were subjected to western blotting to determine the amount of PKCι and β actin with the respective antibodies. Densitometric analysis was performed to obtain relative level of PKCι in each cell line. **e** Linear regression was used to calculate the correlation between *PRKCI* amplification and PKCι protein abundance in ovarian cancer cell lines. Level of PKCι protein was normalized to β-actin protein abundance

To substantiate the results generated from publicly available datasets, we performed our own analysis on 12 ovarian cancer cell lines. Copy number analysis showed that CAOV-3, OCC1, OVCAR3, OVCAR4, OVCA433, and SK-OV3 were *PRKCI*-amplified while other lines were not (Fig. 2a). RT-qPCR showed that *PRKCI*-amplified lines generally displayed higher level of PKCι mRNA (Fig. 2b) and there was good correlation between *PRKCI* copy number and PKCι mRNA (Fig. 2c). Although western blotting showed an overall correlation between *PRKCI* amplification and the abundance of PKCι protein (Fig. 2d, e), we did detect some exceptions. For examples, level of PKCι protein in non-*PRKCI*-amplified HEY was higher than in *PRKCI*-amplified OCC1 (Fig. 2d).

### Silencing PKCι specifically inhibits growth of ovarian cancer cells with *PRKCI* amplification

To investigate whether the status of *PRKCI* amplification was linked to tumorigenic behaviors of ovarian cancer cell lines, we examined growth and migration on both *PRKCI*-amplified or non-amplified lines. MTT assay showed an increase of ~80–290% in 3-day growth period among these cell lines with no significant difference between two groups (Supplementary Data Fig. S1a). Transwell assay also did not reveal apparent association between *PRKCI* amplification and migratory behavior (Supplementary Data Fig. S1b). Similarly, bioinformatics analysis of TCGA dataset did not show significant difference in overall survival or recurrence-free survival between ovarian patients with and without *PRKCI* amplification (Supplementary Data Fig. S1c, d).

**Fig. 3:**
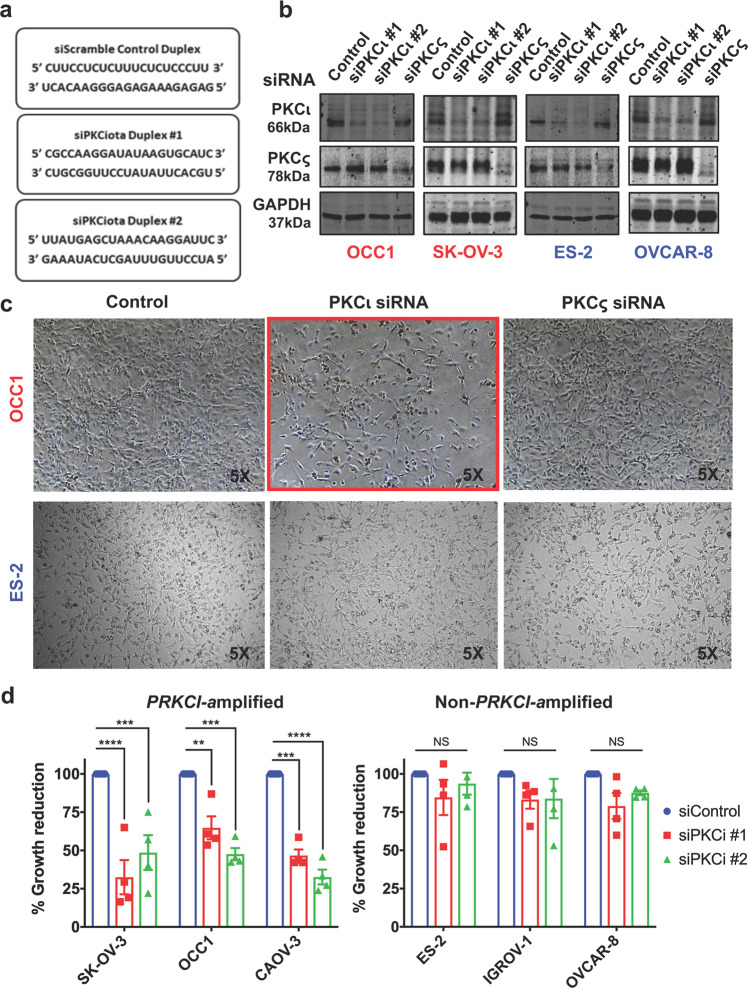
Silencing PKCι selectively leads to growth inhibition in *PRKCI*-amplified ovarian cancer cells. **a** Sequences of two siRNA duplexes against PKCι mRNA and a Scramble siRNA control duplex (purchased from Integrated DNA Technologies). **b***PRKCI*-amplified cell lines SK-OV-3 and OCC1 and non-*PRKCI*-amplified cell lines OVCAR-8 and ES-2 were transfected with 50 nM siRNA for 4 days followed by western blotting to detect PKCι, PKCς, and β actin with the respective antibodies. **c** Images of cells taken four days after transfection of 50 nM control, PKCι or PKCς siRNA. **d** Cells were transfected with 50 nM control or PKCι siRNA for overnight and then reseeded in 24-well to grow for 4 days. The percent growth reduction was compared relative to control treated samples, which were normalized to 100%. Data are means ± SEM. *n* = 4. **P < 0.01; ***P < 0.001; ****P < 0.0001; NS indicates no significance determined by unpaired *t* test with Welch’s correction

The failure to establish a correlation between the status of *PRKCI* amplification and ovarian cancer malignancies prompted us to test the hypothesis that PKCι is differentially required between *PRKCI*-amplified and non-amplified ovarian cancer, and only *PRKCI*-amplified ovarian cancer cells are addicted to the function of PKCι. To test this hypothesis, we introduced PKCι siRNAs into both *PRKCI*-amplified and non-amplified ovarian cancer cell lines (sequences in Fig. 3a) and their silencing effect was confirmed by western blotting (Fig. 3b). Microscopic observation showed that silencing PKCι led to a significant reduction in cell numbers in *PRKCI*-amplified OCC1, CAOV-3, and SK-OV3 cells but not in non-*PRKCI*-amplified ES2, IGROV-1 and OVCAR8 cells (Fig. 3c and Supplementary Data Fig. S2). Further MTT assays showed a trend that growth of *PRKCI*-amplified cell lines is greatly inhibited by knockdown of PKCι whereas silencing PKCι displayed little effect in growth of non-*PRKCI*-amplified lines (Fig. 3d). Since knockdown of PKCι was well tolerated in both OSE and FTEC (Supplementary Data Fig. S3) and silencing PKCς did not exhibit obvious growth-suppressive effect of ovarian cancer cells (Fig. 3c), these results demonstrate a specific requirement for PKCι in growth of *PRKCI*-amplified ovarian cancer cells. In addition, we noticed that non-*PRKCI*-amplified IGROV1 line was insensitive to PKCι knockdown although PKCι protein abundance in this line was similar to that in *PRKCI*-amplified OCC1 (Fig. 2d). These results indicate that the status of *PRKCI* amplification rather than level of PKCι protein determines the addiction to PKCι in ovarian cancer cells.

### *PRKCI-*amplified ovarian cancer cells undergo apoptosis upon depletion of PKCι

**Fig. 4:**
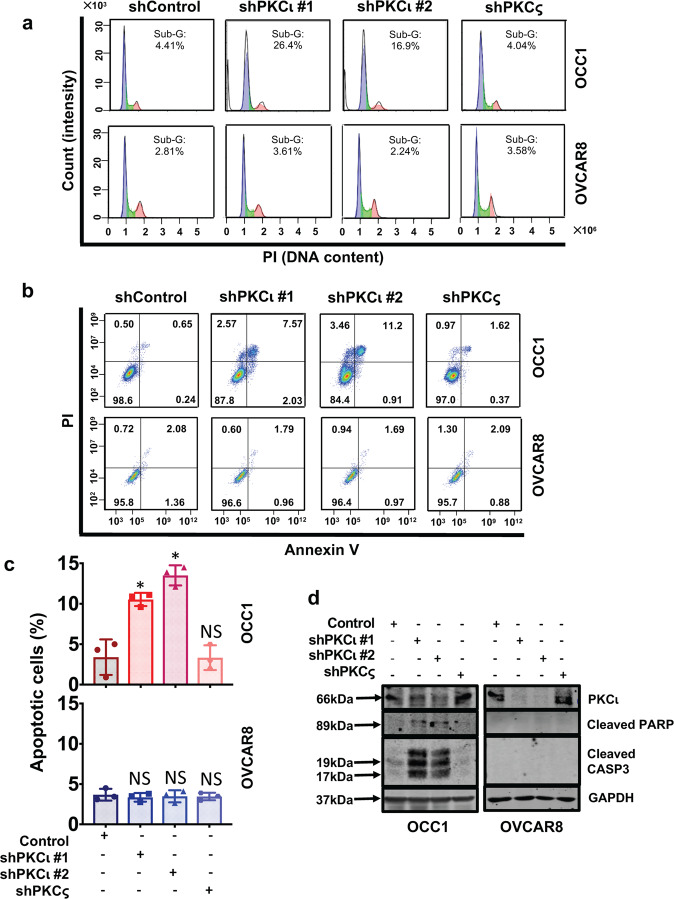
Knockdown of PKCι induces apoptosis in *PRKCI*-amplified ovarian cancer cells. **a** Ovarian cancer cells were infected with lentiviral vector containing Scramble, PKCι or PKCς shRNA for 4 days followed by flow cytometry to analyze cell cycle progression. The horizontal axis is represented by PI (DNA content) and the vertical axis is represented by Count (Intensity). Results are representative of three independent experiments. **b** Ovarian cancer cells were infected with lentiviral vector containing Scramble, PKCι or PKCς shRNA for 4 days and then subjected to Annexin V/PI-based flow cytometry. The horizontal axis is represented by Annexin V and the vertical axis is represented by PI. Cells in Quadrant 2 and 3 represent apoptotic cells. Results are representative of three independent experiments. **c** Quantitation of results from the Annexin V/PI analysis. Data are means ± SEM. *n* = 3. *P < 0.01 versus control, determined by unpaired *t* test with Welch’s correction. NS indicates no significance. **d** Ovarian cancer cells were infected with lentiviral vector containing Scramble, PKCι or PKCς shRNA for 4 days followed by western blotting to detect PKCι, cleaved PARP, cleaved CASP3 and GAPDH with the respective antibodies

To explore the cause of PKCι knockdown-led growth reduction in *PRKCI*-amplified ovarian cancer cells, we initially analyzed cell cycle progression of cells with PKCι or PKCς knockdown. Flow cytometry showed that silencing PKCι, but not PKCς led to a dramatic increase in Sub-G1 population in both *PRKCI*-amplified OCC1 and SK-OV3 cells (Fig. 4a and Supplementary Data S4a). In contrast, such increase was not detected in non-*PRKCI*-amplified ES2 and OVCAR8 cells (Fig. 4a and Supplementary Data Fig. S4a). The detection of increased Sub-G1 population prodded us to investigate the possibility of apoptosis upon PKCι knockdown. Annexin V/PI-based flow analysis showed that there were significantly higher levels of apoptotic cells in the *PRKCI*-amplified ovarian cells than in non-*PRKCI*-amplified ones after PKCι knockdown (Fig. 4b, c and Supplementary Data Fig. S4b). Western blotting further confirmed the occurrence of apoptosis in *PRKCI*-amplified ovarian cancer cells upon the condition of PKCι knockdown, as evidenced by the appearance of cleaved PARP and cleaved CASP3 (Fig. 4d and Supplementary Data Fig. S5). In addition, this apoptotic cell death was PKCι isoform specific as PKCς knockdown did not affect cell viability in any of the cell lines (Fig. 4b–d, Supplementary Data Figs. S4b, S5). Our results demonstrate that that silencing PKCι selectively induces apoptosis in *PRKCI*-amplified ovarian cancer cells while having little effect on non-*PRKCI*-amplified cells.

We next investigated whether increasing copies of *PRKCI* gene could confer addiction to PKCι in non-*PRKCI*-amplified ovarian cancer cells by lentivirally introducing *PRKCI* into OVCA429 cells (Supplementary Data Fig. S6a). However, OVCA429 cells with ectopic PKCι expression remained insensitive to PKCι knockdown as indicated by lack of cleaved CASP3 (Supplementary Data Fig. S6b). These results were consistent with notion that only lines with intrinsic *PRKCI* amplification are addicted because of the nature of *PRKCI* amplification as an oncogenic driver while non-*PRKCI*-amplified ovarian cancer cells are driven by other oncogenic drivers and thus not dependent on the presence of PKCι even PKCι was ectopically overexpressed.

### EpCAM aptamer-delivered PKCι siRNA effectively induces apoptosis in *PRKCI*-amplified ovarian cancer cells

The observation that silencing PKCι leads to apoptosis in *PRKCI*-amplified ovarian cancer cells urged us to turn attention on those small molecules reported to inhibit atypical PKCs such as Aurothiomalate (ATM), Auranofin (ANF) and Oncrasin-1 as potential therapeutic agents for the subset of ovarian cancer patients with *PRKCI* amplification. ATM and ANF were previously reported to block atypical PKC signaling by blocking the interaction between atypical PKCs and Par6, Ect2, or p62^10,22^ while Oncrasin-1 was found to only induce cell death in the presence of both mutant K-Ras and PKCι.^23^ However, MTT assay showed that the IC_50_ values calculated for ATM and Oncrasin were quite high compared to the published values for lung cancer. For example, SK-OV3 cells had an IC_50_ of 84.9 µM for ATM and an IC_50_ of 182.8 µM for Oncrasin-1 (Supplementary Data Fig. S7), despite it being a *PRKCI*-amplified cell line. ANF did present lower IC_50_ values compared to the other two inhibitors but there was no specificity noted for *PRKCI*-amplified cell lines (Supplementary Data Fig. S7). To make the situation worse, we observed that 1 µM ANF displayed high toxicity in FTEC cells (Supplementary Data Fig. S8). These results thus rule out the potential of using these inhibitors as therapeutic option for the subset of ovarian cancer patients with *PRKCI* amplification.

**Fig. 5:**
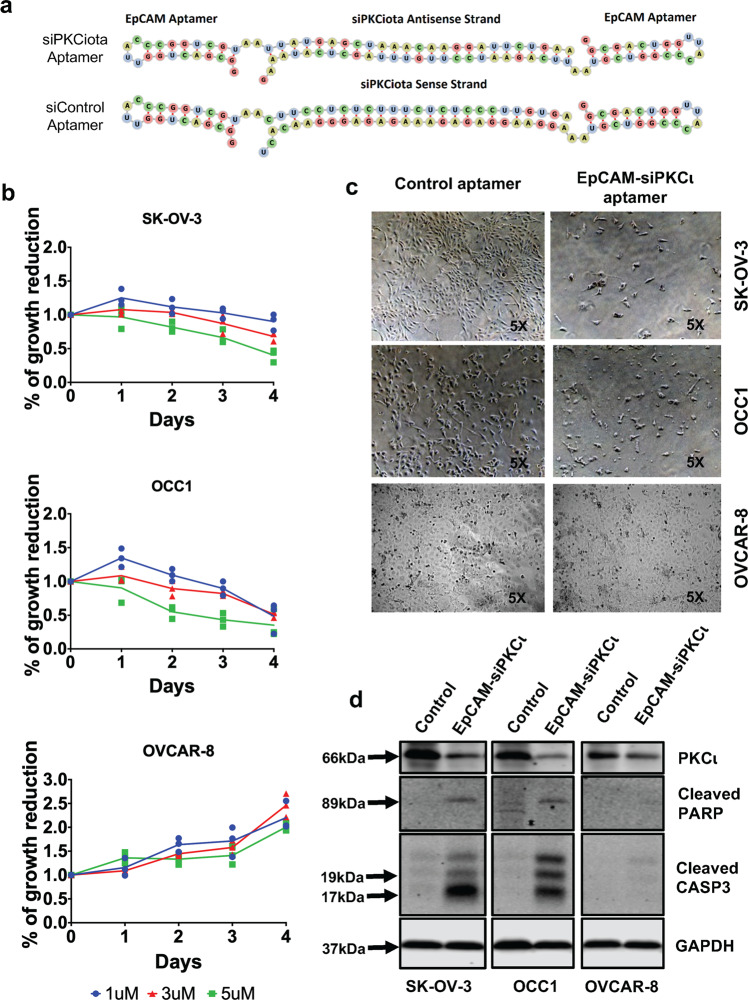
EpCAM aptamer-delivered PKCι siRNA triggers apoptosis in *PRKCI*-amplified ovarian caner cells. **a** Structure of EpCAM-PKCι siRNA chimera. **b** SK-OV3, OCC1 and OVCAR8 cells were treated with varying concentration of EpCAM-siPKCι aptamer up to 4 days followed by MTT assay. Absorbance readings at the 12 h were marked as Day 0 and all other time points were normalized to this value to calculate percentage growth. Data are means ± SEM. *n* = 3. **c** Cells were treated with 5 µM control or EpCAM-PKCι aptamer for 4 days followed by imaging under the microscope using 5X objective. **d** Cells were treated with 5 µM control or EpCAM-PKCι aptamer for 4 days, and then harvested for western blotting to detect PKCι, cleaved PARP, cleaved CASP3 and GAPDH with the respective antibodies

We next investigated the possibility to use a known 20-nucleotide long EpCAM ssRNA aptamer to deliver PKCι siRNA into ovarian cancer cells based on the knowledge that this EpCAM aptamer not only processes similar binding affinity as antibodies but is also efficiently internalized into cells through receptor-mediated endocytosis.^24^ Especially, elevated level of EpCAM is detected in over 70% ovarian cancer and its expression in the peritoneal cavity is tumor specific.^20,21^ Toward this end, we designed an AsiC (called as EpCAM-siPKCι aptamer) in which both ends are the EpCAM aptamers and the middle portion is the sense and antisense sequences for PKCι siRNA or scramble sequence (Fig. 5a). We included two unpaired “A”s which serve as spacer sequences to mediate the flexibility and successful recognition of the aptamer as it progresses from receptor-mediated endocytosis to eventual Dicer processing (Fig. 5a). We also incorporated 2’fluoro (F)-pyrimidines into the aptamer during the in vitro transcription stage in order to promote stability and prevent nuclease-mediated degradation of the annealed siRNA-aptamer.^17^ To illustrate the effect of EpCAM aptamer-delivered PKCι siRNA on cell growth, we treated SK-OV3, OCC1 and OVCAR8 cells with varying doses of Control or EpCAM-siPKCι aptamer for different length of times. MTT assay showed that response to the increased concentration of EpCAM-siPKCι aptamer, but not the control aptamer, led to decrease in cell growth in *PRKCI*-amplified SK-OV3 and OCC1 cells. In contrast, growth of OVCAR-8, the non-*PRKCI*-amplified cell line, was not affected by EpCAM-siPKCι aptamer (Fig. 5b). Morphologically, SK-OV-3 and OCC1 cells could be seen dying and in remarkably reduced number when treated with EpCAM-siPKCι aptamer (Fig. 5c). In contrast, EpCAM-siPKCι aptamer was well tolerated by OVCAR-8 cells (Fig. 5c). EpCAM-siPKCι aptamer-led cell growth reduction in *PRKCI*-amplified ovarian cancer cells was clearly caused by apoptosis as we detected apparent cleavage of PARP and CASP3 after 3-day treatment in SK-OV-3 and OCC1 but not in the OVCAR-8 cell line (Fig. 5d). These results validate the usefulness as well as efficacy of EpCAM aptamer to deliver siRNA into ovarian cancer cells.

### EpCAM-siPKCι aptamer effectively suppresses intraperitoneal ovarian cancer development

**Fig. 6:**
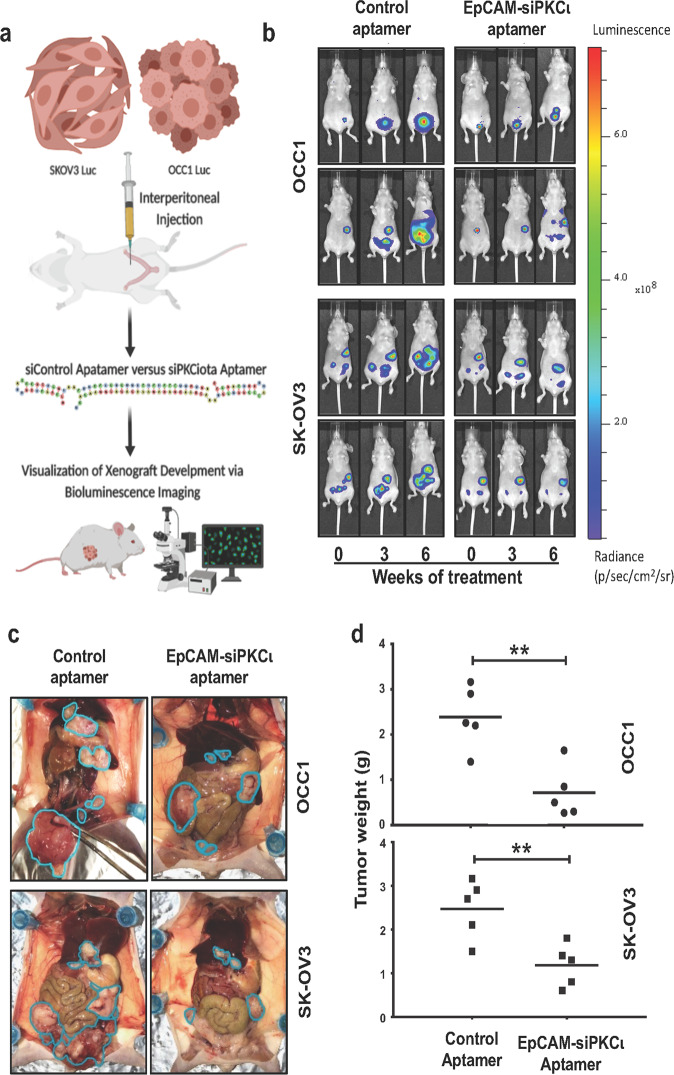
EpCAM aptamer-delivered PKCι siRNA suppresses intraperitoneal xenograft development. **a** Scheme of experimental procedure. Luciferase-expressing SK-OV-3 or OCC1 cells (10^7^ cells/mouse) were intraperitoneally inject into athymic nude mice. Once tumors were detected, mice were divided into two groups: one was treated with control aptamer and the other with EpCAM-siPKCι aptamer. Xenograft development was monitored using the IVIS bioluminescence imaging system. **b** Images of the xenograft tumors using the Xenogen IVIS at point of treatment (marked 0), 3 weeks and 6 weeks of treatment. Two representative mice from each group were included. The image data is displayed in radiance or photons/sec/cm^2^/steradian. **c** Mice were sacrificed after 7 weeks of treatment and images were taken after dissection. Tumor are circled in teal. **d** Tumor implants were collected and weighed. Average of tumor weights for each group were calculated (*n* = 5). ** indicates *P* < 0.01 EpCAM-siPKCι aptamer versus control determined by ANOVA and student *t* tests

The finding that EpCAM aptamer-delivered PKCι siRNA selectively induces apoptosis in *PRKCI*-amplified ovarian cancer cells led us to further investigate the efficacy of EpCAM-siPKCι aptamer to suppress ovarian tumor progression. Female athymic nude mice were intraperitoneally injected with luciferase-expressing OCC1 or SK-OV3 cells and administration of Control and EpCAM-siPKCι aptamer at 200nmole/mouse began as soon as tumor was detected (about 1 week after intraperitoneal injection of 10^7^ cells/animal). Treatment was carried out three times a week and tumor outgrowth was monitored weekly (Fig. 6a). Bioluminescence imaging showed that intraperitoneal xenograft of both cell lines developed rapidly in mice receiving Control aptamer (Fig. 6b and Supplementary Data Fig. S9). In contrast, administrating EpCAM-siPKCι aptamer deterred tumor propagation (Fig. 6b and Supplementary Data Fig. S9). At 7 weeks post-treatment, all mice were euthanized and intraperitoneal tumor development was visualized. We observed that tumor implants were much less and smaller in mice receiving EpCAM-siPKCι aptamer than those seen in mice administered with Control aptamer (Fig. 6c). To quantitate the difference, we harvested tumor implants from sacrificed mice. Tumor weights were significantly lower in EpCAM-siPKCι aptamer-treated mice than Control aptamer-treated ones in both OCC1 and SK-OV3 cells (Fig. 6d). In a parallel, weights of the mice were measured every week during treatment. No significant differences were noticed when control and EpCAM-siPKCι aptamer- treated mice were compared (Supplementary Data Fig. S10), indicating that EpCAM aptamer-delivered siRNA does not induce severe toxicity to mice.

To link deterred tumor development to diminished PKCɩ expression and apoptosis, we performed IHC to examine the intensity of PKCɩ, cleaved CASP and TUNEL staining on harvested tumors. Strong PKCɩ but no cleaved CASP3 or TUNEL were detected in tumors excised from control aptamer-treated mice (Supplementary Data Fig. S11a). In contrast, tumors derived from EpCAM-siPKCɩ aptamer-treated mice displayed little PKCɩ but robust cleaved CASP3 and TUNEL staining (Supplementary Data Fig. S11a). In a parallel experiment, we determined the effect of EpCAM-siPKCɩ aptamer on survival of mice bearing OCC1 tumors. Mice were injected with OCC1 cells for 1 week and then administrated with control or EpCAM-siPKCɩ aptamer. The earliest moribund of tumor-bearing mice receiving control aptamer occurred 2-week posttreatment and all mice died within 5 weeks (Supplementary Data Fig. S11b). However, treatment of EpCAM-siPKCɩ aptamer significantly increased the lifespan of mice receiving OCC1 cells (*p* < 0.005) (Supplementary Data Fig. S11b). These results support the notion that EpCAM-siPKCɩ aptamer suppresses tumor development and prolong survival of tumor-bearing mice by depleting PKCɩ and inducing apoptosis.

## Discussion

PKCι is highly expressed in diverse cancer types through various mechanisms including increased transcription and translation.^25^ In ovarian cancer, *PRKCI*, the gene for PKCι is amplified in over 30% patients (Fig. 1a). *PRKCI* amplification also translates to elevated levels of PKCι mRNA and protein both in ovarian cancer specimens and ovarian cancer cell lines (Figs. 1 and 2). The fact that *PRKCI* is the only PKC family member highly amplified in ovarian cancer (Fig. 1) indicates that PKCι plays a critical role in ovary tumorigenesis. This notion is supported by recent findings that PKCι maintains tumor-initiating cell phenotype, enhances YAP1 activity and augment immune suppression in ovarian cancer.^10–12^

Despite over 30% of ovarian cancer patients harbor *PRKCI* amplification and reported tumor-promoting role of PKCι in experimental ovarian cancer models, we failed to detect clear association between the status of *PRKCI* amplification and survivals of ovarian cancer patients (Supplementary Data Fig. S1). Analyzing established ovarian cancer cell lines also did not reveal significant difference in cell growth and migration between *PRKCI*-amplified and non-amplified cell lines (Supplementary Data Fig. S1). Such apparent discrepancy led us to hypothesize that PKCι is differentially involved in ovary tumorigenesis depending on *PRKCI* amplification status. This hypothesis is based on the known fact that CNAs can sometimes provide oncogenic drivers, such as *HER2* amplification in breast cancer^26^ and *CCNE1* in ovarian cancer.^27^ In this study, we observed that silencing PKCι induced apoptosis in *PRKCI*-amplified ovarian cancer cells while it was well tolerated by cells without *PRKCI* amplification even though these lines displayed even greater abundance of PKCι (Figs. 3 and 4). This observation led us to believe that *PRKCI* amplification is an oncogenic driver and ovarian cancer cells with *PRKCI* amplification are addicted to the presence of PKCι expression regardless its abundance. Contrarily, oncogenic etiologies of non-*PRKCI*-amplified ovarian cancer cells are not associated with *PRKCI* amplification and their survivals are thus independent on PKCι expression even they might have elevated level of PKCι.

Oncogene addiction is a phenomenon that growth/survival of cancer cells necessitates an activation of oncogene or inactivation of tumor suppressor gene.^28^ This phenomenon has allowed the successful development of precision medicine-based molecular target therapy. For example, the HER2-targeting trastuzumab (Herceptin), which markedly inhibits tumor growth and prolongs survival of *HER2*-amplified breast cancer patients.^29^ The use of EGFR antagonists including gefitinib and erlotinib in lung cancer treatment is another example of oncogene addiction that has led to success in a subset of patients with mutations in the kinase domain of EGFR.^30^ Our observed addiction to PKCι in *PRKCI*-amplified ovarian cancer cells suggest that a precision medicine-based therapeutic approach can be developed against a subset of ovarian cancer patients with *PRKCI* amplification. Unfortunately, we found that those reported small molecule inhibitors of atypical PKC (ATM, ANF and Oncrasin) were either not selective to *PRKCI*-amplified ovarian cancer cells or cytotoxic to non-cancerous FTSE cells at effective doses. Therefore, we were forced to envision a different approach to interfere with PKCɩ functionality and reasoned that PKCι siRNA could be an ideal agent to achieve this goal. As EpCAM is highly expressed in ovarian cancer but not in other tissues in peritoneal cavity,^20,21^ we took advantage of a well characterized EpCAM aptamer as the vehicle for PKCι siRNA delivery. With this AsiC (EpCAM-siPKCι aptamer), we achieved efficient knockdown of PKCι in ovarian cancer cells and detected dose-dependent apoptosis only in *PRKCI*-amplified ovarian cancer cells (Fig. 5). Importantly, we showed that EpCAM-siPKCι aptamer suppressed intraperitoneal tumor development of *PRKCI*-amplified ovarian cancer cells and prolonged lifespan of tumor-bearing mice (Fig. 6). It is worth pointing out that our hypothesis was initially derived from available HGSOC data available from TCGA while OCC1 is a clear cell ovarian cancer cell line. These results indicate that *PRKCI* amplification may be an oncogenic driver not only limited to HGSOC.

Our study demonstrates that ovarian cancer cells with *PRKCI* amplification undergo apoptosis at a higher rate when PKCι is silenced, indicating that *PRKCI* amplification is a critical factor driving ovary tumorigenesis and such cells are thus addicted to PKCι function. Although cancer cell lines in culture are imperfect models of human tumors, they tend to remain addicted to the factors that initiated tumor formation and hence are well-validated tools for studying precision medicine-based target therapies.^31^ Therefore, our study suggest that PKCι is an ideal therapeutic target for a subset of ovarian cancer with *PRKCI* amplification and has laid a foundation on using EpCAM aptamer-delivered PKCι siRNA to treat this subset of ovarian cancer.

## Materials and methods

### Cell culture and other reagents

All cell lines were cultured in DMEM supplemented with 10% fetal bovine serum in a humidified incubator containing 5% CO2 at 37 °C. Cell culture media for OSE and FTECs consists of a 1:1 ratio of MCDB 105 and Medium 199 supplemented with 10 ng/mL epidermal growth factor. Aurothiomalate (ATM) and Auronofin (ANF) were purchased from Sigma-Aldrich (St. Louis, MO) while Oncrasin-1 was obtained from Tocris Bioscience (Bristol, United Kingdom). PKCι siRNAs and scrambled control were purchased from Integrated DNA Technologies (Coralville, IA) and PKCς siRNA pool was obtained from Dharmacon (Lafayette, CO).

### Copy number analysis

PCR fragments of *PRKCI* and *RPP1* (Ribonuclease P/MRP protein subunit) were generated and used to generate a standard curve for *PRKCI* and *RPP1* using qPCR. Primer set (forward/reverse) for *PRKCI* is ACCCTTCATACGAAGTGCACAA/TCCCCCATCAAACTGCTTCTC. Primer set for *RPP1* is CTTGTGGGTGGTGCCATTTG/GGTCAATCGCCTTCACAGGA. To determine *PRKCI* copy number, genomic DNA was collected for 12 ovarian cancer cell lines, OSE, FTEC, lung cancer A549, and breast surface epithelial cell line MCF10A1. Values of *PRKCI* and *RPP1* cycle threshold (Ct) were computed via qPCR using the genomic DNA. The values were then compared to the standard curves and the ratios were also compared to OSE and FTEC (which do not have *PRKCI* amplification). TaqMan Copy Number Assay (Invitrogen, Carlsbad, CA) was additionally used to analyze the genomic DNA of the ovarian cancer cell lines to confirm the results from the standard curve approach. OSE line (T29) was used as the calibrator sample, and all samples were normalized to *RPP1*.

### RNA isolation and RT-qPCR

Total RNA was collected using Trizol, treated with DNaseI and used to generate cDNA. Generated cDNA was subjected to RT-qPCR to measure PKCι and βActin mRNA levels. The level of PKCι was standardized by comparing its Ct values to that of βActin. RT-qPCR primer set for PKCi is – forward: AGGTCCGGGTGAAAGCCTA; reverse: TGAAGAGCTGTTCGTTGTCAAA. QRT-PCR primer set for βActin are – forward: CCAGCTCACCATGGATGATG; reverse: ATGCCGGAGCCGTTGTC.

### Western blot analysis

Cell lysates were prepared using radio-immunoprecipitation assay buffer supplemented with protease and phosphatase inhibitors (Bimake, Houston, TX). Equal amounts of protein were loaded per lane into an SDS-PAGE followed by transferring onto a nitrocellulose membrane (Invitrogen). The blots were blocked with 5% nonfat dried milk followed by incubation in the respective antibodies. After several washes, membranes were incubated with secondary infrared antibodies and imaged using the LICOR Odyssey Infrared Imaging System (Lincoln, NE). A list of antibodies used is included in the Supplementary Data Table S1. To determine the effect of PKCι knockdown on apoptosis, cells were either infected with lentivirus containing PKCι shRNA for 4 days or treated with 5 µM EpCAM-siPKCι for 4 days. Cells were then lysed and cell lysates subjected to western blotting to detect cleaved PARP and CASP3 with the respective antibodies.

### Cell growth assay

Cell growth was analyzed by MTT [3-(4,5-dimethylthiazol-2-yl)-2,5-diphenyltetrazolium bromide] colorimetric assay as described previously.^32^ Briefly, cells were seeded in two 48-well plates (4000 cells/well) in triplicates. MTT assay was performed at two time points: 12 h (Day 0) and 84 h after seeding (Day 3). The relative cell growth was expressed as a percentage relative to Day 0 values. To determine the effect of PKCι knockdown on cell growth, cells were either transfected with 50 nM PKCι siRNA or treated with varying concentration of aptamer for 4 days followed by MTT assay.

### Migration assay

Cell migration was assessed by Transwell as previously described.^33^ Briefly, the undersurface of upper chamber (8.0 mm pore size) was coated with 10 µg/mL of collagen I and 500 µL of serum-free media was added to the lower chambers. Upper chambers were placed inside the lower chambers and 100,000 cells were pipetted into each upper chamber. After 4 h, cells in upper chambers were removed using cotton swabs while cells on undersurface were fixed in 4% paraformaldehyde for 30 min and then stained with crystal violet solution. Cells were counted and quantified using the Imaris 7.0 imaging software to compute the number of voxels (volumetric pixel). To determine the effect of PKCι knockdown on cell migration, cells were transfected with 50 nM PKCι siRNA for 4 days followed by Transwell assay.

### Flow cytometry to analyze cell cycle progression and apoptosis

The effect pf PKCι knockdown on cell cycle progression was determined as previously described.^34,35^ Briefly, cells were infected with lentivirus containing PKCι shRNA for 4 days, detached with trypsin and washed thoroughly. After fixing cells in 66% ethanol, cells were suspended in PBS containing 20 μg/mL of propidium iodide (PI) and subjected to flow cytometry using BD Accuri C6 Plus flow cytometer (BD Biosciences, Bedford, MA). Generated data were analyzed using the BD FACSDiva Software. Extent of apoptosis was assessed using the FITC-Annexin V/PI detection kit (BD Biosciences) as previously described.^35,36^ The percentage of apoptotic cell population was determined using the ModFit LT 3.0 software (BD Biosciences).

### Generation of EpCAM-siPKCι aptamer

Aptamers were generated through in vitro transcribed RNA transcripts as previously described.^37^ Detailed information on the oligonucleotides (T7, PKCι siRNA and scrambled oligo) synthesized for in vitro transcription is provided in Supplemental Data. The T7 Promoter sequence was annealed with PKCι siRNA or scrambled oligonucleotide. Generated partially single-stranded templates were then subjected to in vitro transcription using Mega in vitro Transcription kit (Promega, Madison, WI). To stabilize RNA transcripts, 2’fluoro (F)-pyrimidines (TriLink Biotechnologies, Dan Diego, CA) were added in a (1:4) ratio in reaction In vitro transcribed transcripts were annealed to generate aptamers. The structures of each aptamer was analyzed using Forna package (http://rna.tbi.univie.ac.at/forna/).

### Mouse xenograft models, imaging, and drug administration

Luciferase-containing OCC1 and SK-OV3 (1 × 10^7^ cells/mouse) were intraperitoneally injected into 5–6-week-old nude female mice (Nude-Foxn1nu, Envigo, Huntingdon, United Kingdom). Intraperitoneal xenograft development was measured by luciferase activity using the Xenogen IVIS-200 In Vivo bioluminescence imaging system. Once tumor was detected in the mice, they were divided into two groups (5 per group) and received either EpCAM-control or EpCAM-siPKCι aptamer thrice a week intraperitoneally (200 nmole/mouse). Tumor outgrowth was monitored weekly. After 6 weeks of treatment, visible implants were collected from peritoneal cavities of euthanized animals for weight measurement and immunohistochemistry staining. All procedures were approved by the Institution Animal Care Committee at University of Florida.

### Immunohistochemistry

Tumor tissues were excised from euthanized mice and paraffin-embedded. Tissue were then sectioned and subjected to immunohistochemistry (IHC) to detect PKCι, Ki67, and CASP3 as well as for TUNNEL staining as previously described.^33^

### Bioinformatics analysis

Gene-level copy number estimation was made using the GISTIC2 method using the TCGA ovarian serous cystadenocarcinoma dataset containing 579 patients (TCGA_OV_gistic2thd-2015–02–24). Values to −2,−1, 0, 1, and 2 represent homozygous deletion, single copy deletion, diploid normal copy, low-level copy number amplification, or high-level copy number amplification, respectively. Kaplan–Meier plotters were drawn using SPSS software. Two groups of patients were split by present or absent amplification of *PRKCI*. Statistical difference of PKCι expression level in *PRKCI* amplification versus the rest was calculated by 2-tailed Student *t* test. Correlation between the level of PKCι expression and magnitude of *PRKCI* amplification were shown in box-and-whisker plots. Linear regressions of gene amplification number versus expression level were calculated using Pearson’s correlation coefficient χ2-tests. *P* < 0.05 was considered statistically significant. *R*^2^ represents the Coefficient of Determination. Dataset for PKCι expression was downloaded from https://tcga.xenahubs.net/download/TCGA.OV.sampleMap/HiSeqV2.gz.

### Statistical analysis

All experiments were performed at least in duplicate. The results of each experiment are reported as the mean of experimental replicates. Error bars represent the Standard Error of Mean (S.E.M), unless otherwise stated. Statistical analyses of cell growth, PKCι mRNA levels, and tumor weights were performed by ANOVA and Student *t* tests. Pairwise comparisons were analyzed using the unpaired *t* test with Welch’s correction (not assuming for equal SDs) to determine significance between control and knockdown of PKCι. Mann–Whitney *U*-test (two-sided) was performed to analyze the statistical difference in cell growth migration between *PRKCI*-amplified and non-amplified cell lines. Linear regression curves were plotted on Microsoft Excel using the x-y scatter graph function. For all tests, *P* < 0.05 was considered significant.

## Supplementary information

Supplemental Materials

## Data Availability

All the data support the findings in this study can be found within the article and its supplementary data. They also can be requested by contacting corresponding authors.
