# Supplementary material for: Addiction to protein kinase Cɩ due to PRKCI gene amplification can be exploited for an aptamer-based targeted therapy in ovarian cancer
Source: Signal Transduct Target Ther. 2020 Aug 21;5:140. doi: 10.1038/s41392-020-0197-8 (PMC7441162; doi:10.1038/s41392-020-0197-8)
Supplement: Supplementary file 1 — Supplemental Materials [file 41392_2020_197_MOESM1_ESM.pdf]

SUPPLEMENTAL INFORMATION AND DATA FOR

Addiction to Protein Kinase Cι due to PRKCI Gene Amplification can be exploited for an Aptamer-Based Targeted Therapy in Ovarian Cancer

Hina Rehmani, Yue Li, Tao Li, Ravi Padia, Ozlem Calbay, Lingtao Jin, Huijun Chen, Shuang Huang

CORRESPONDENCE TO:
shuanghuang@ufl.edu
karrel@sina.com.

This PDF file includes:

FIGURES. S1 TO S11
TABLES S1

SUPPLEMENTARY INFORMATION AND DATA

Oligonucleotide Sequences for Aptamers

The siRNA sequence for Scramble Control (25/27mer) is the following:

Forward: 5’ CUUCCUCUCUUUCUCUCCCUUGUGA 3’

Reverse: 3’ UCACAAGGGAGAGAAAGAGAGGAAGGA 5’

The siRNA sequence against PKCι (25/27mer) is the following:

Forward: 5’ UUAUGAGCUAAACAAGGAUUCUGAA 3’

Reverse: 3’ GAAAUACUCGAUUUGUUCCUAAGACUU 5’

Partially Single-Stranded Oligo Template Design Ordering Info for Aptamers:

T7 Promoter: 5’ AATT**TAATACGACTCACTATAG** 3’

PKCι Oligo 1:

5’ ttcagaatccttgtttagctcataattacgaccgggtaaccagtcgcc**TATAGTGAGTCGTATTAAATT** 3’

PKCι Oligo 2:

5’ ctttatgagctaaacaaggattctgaattacgaccgggtaaccagtcgcc**TATAGTGAGTCGTATTAAATT** 3’

Partially Single-Stranded Oligo Template Design Ordering Info:

Scramble Oligo 1:

5’ tcacaagggagagaaagagaggaagttacgaccgggtaaccagtcgcc**TATAGTGAGTCGTATTAAATT** 3’

Scramble Oligo 2:

5’ agtgttccctctctttctctccttcctttacgaccgggtaaccagtcgcc**TATAGTGAGTCGTATTAAATT** 3’

To anneal synthetic oligonucleotides to make a transcription template, it is only necessary that the promoter sequence of a template be double-stranded. The T7 Promoter sequence was annealed to form a double-stranded promoter using the oligonucleotides listed above. The oligonucleotides were suspended in TES (10mM Tris-HCl, pH 8, 1mM EDTA, 0.1M NaCl) and mixed in equimolar

amounts to a final concentration of ~10-50µM of each oligonucleotide. The mixture was brought to 95°C in a heat block for a few minutes and then allowed to cool to room temperature. Afterwards, these annealed, partially single-stranded templates generated were then used in the *in vitro* transcription kits (Promega). Aptamers were generated using RNA generated from 2 sequences, hence Oligo 1 and Oligo 2 listed above.

Visualization for Aptamers

The Forna package is an RNA secondary structure visualization tool that was used to edit and display the RNA-based structures of the aptamers generated in this study (http://rna.tbi.univie.ac.at/forna/).

Aptamer Synthesis

Control and PKCiota aptamers were individually synthesized by in vitro transcription (Promega) with phage promoter-containing synthetic oligonucleotides as templates (IDT). The oligonucleotides were PAGE or HPLC purified to remove partial sequences. The T7 RNA polymerase promoter is underlined and the EpCAM aptamer is bolded.

Forward Primer for EpCAM (28nt):

5' TAATACGACTCACTATA**GCGACTGGTTA** 3'

ssDNA of EpCAM aptamer (62nt):

5' TAATACGACTCACTATA**GCGACTGGTTACCCGGTCGT** 3'

Each aptamer is composed of two RNA structures that were generated in separate in vitro transcription reactions but then were annealed together in a 1:1 molar ratio. Normal purine and pyrimidines were added to the mixture but 2'fluoro (F)-pyrimidines (TriLink Biotechnologies) were also added in a (1:4) ratio and the reaction mixture was adjusted to compensate for the additional volumes. The two RNAs were annealed to form one entity by heating at 94°C for 3

minutes followed by slowly cooling to room temperature within 1 hour. Annealed aptamers were stored in -80C.

Supplementary Data Figure S1

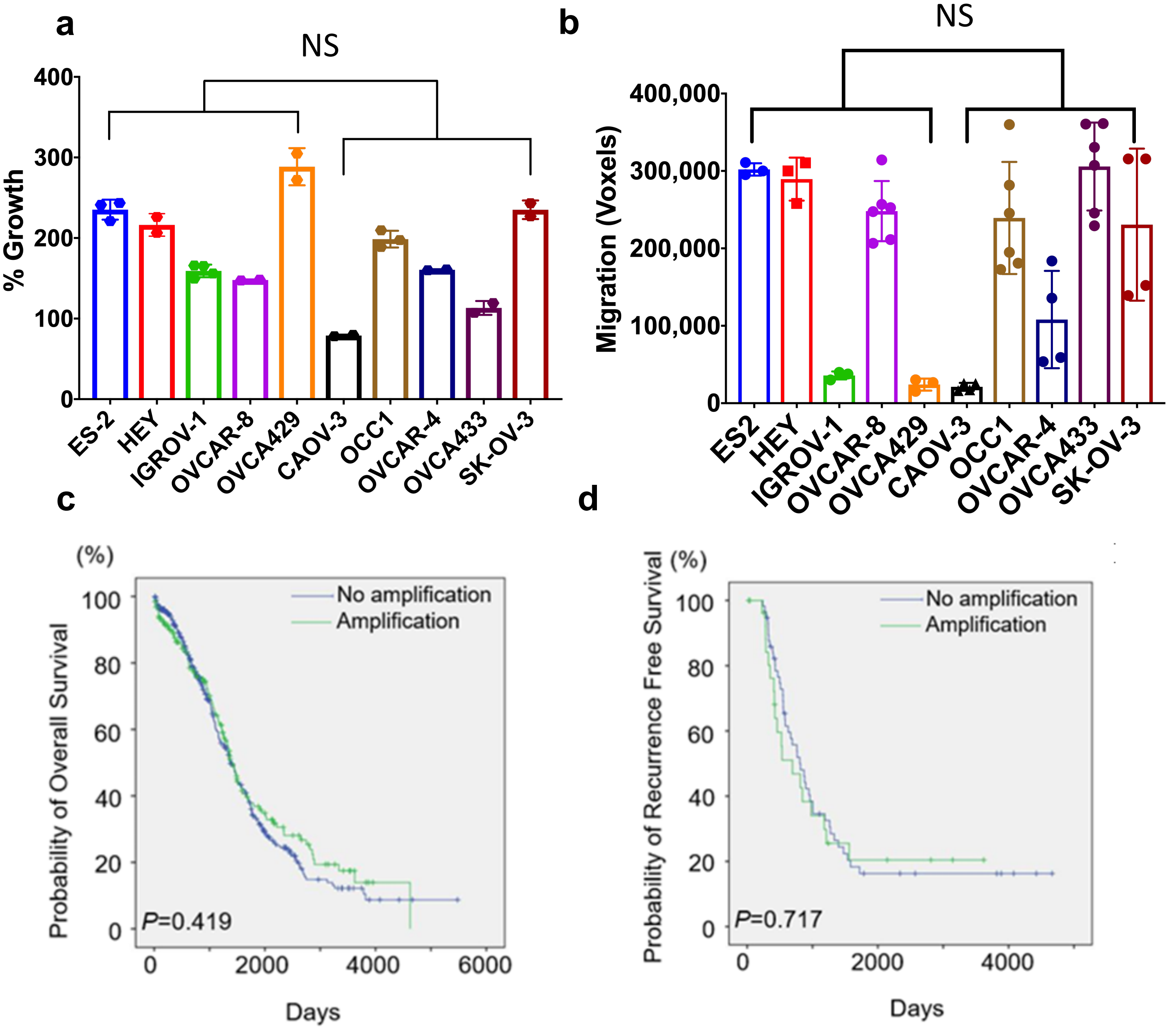

Figure S1. The status of *PRKCI* amplification is not associated with ovarian cancer patient survival or tumorigenic behaviors in established ovarian cancer cells. *a*. Overnight-cultured cells were trypsinized and added into 96-well plates and MTT assay was performed at 12 and 84 h after plating. The percent growth was expressed as OD at 84 h relative to value at 12 h. Data are mean ± SD. Mann-Whitney U-test was used to compare the difference between *PRKCI*-amplified and non-amplified groups. NS indicate no statistical significance. ***b***. Overnight-cultured cells were subjected to Transwell assay. Cells on undersurface of upper chambers were stained and counted using the Imaris 7.0 imaging software. Data are means ± SD. The Mann-Whitney U-test was performed to compare the difference between *PRKCI*-amplified and non-amplified groups. NS indicate no statistical significance ***c***. Kaplan Meier curve of ovarian cancer patients' overall survival. ***d***. Kaplan Meier curve of ovarian cancer patients' recurrence free survival.

Supplementary Data Figure S2

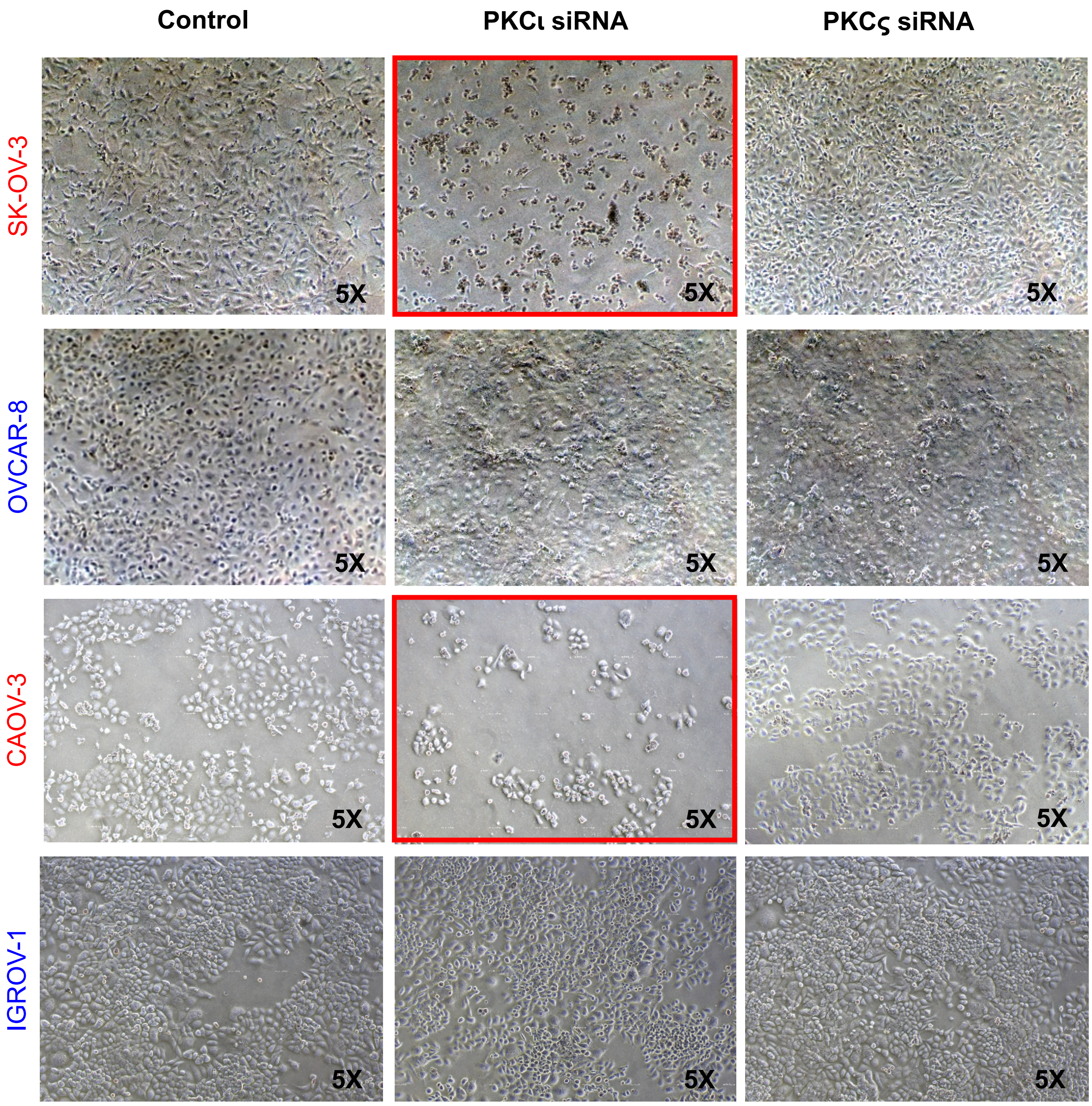

Figure S2. Silencing PKCι selectively inhibits growth of *PRKCI*-amplified ovarian cancer cells. *PRKCI*-amplified cell lines SK-OV-3 and CAOV-3 and non-*PRKCI*-amplified cell lines OVCAR-8 and IGROV-1 were transfected with 50nM siRNA for 4 days. Images of cells were under a phase-contrast microscope after 4 days of transfection.

Supplementary Data Figure S3

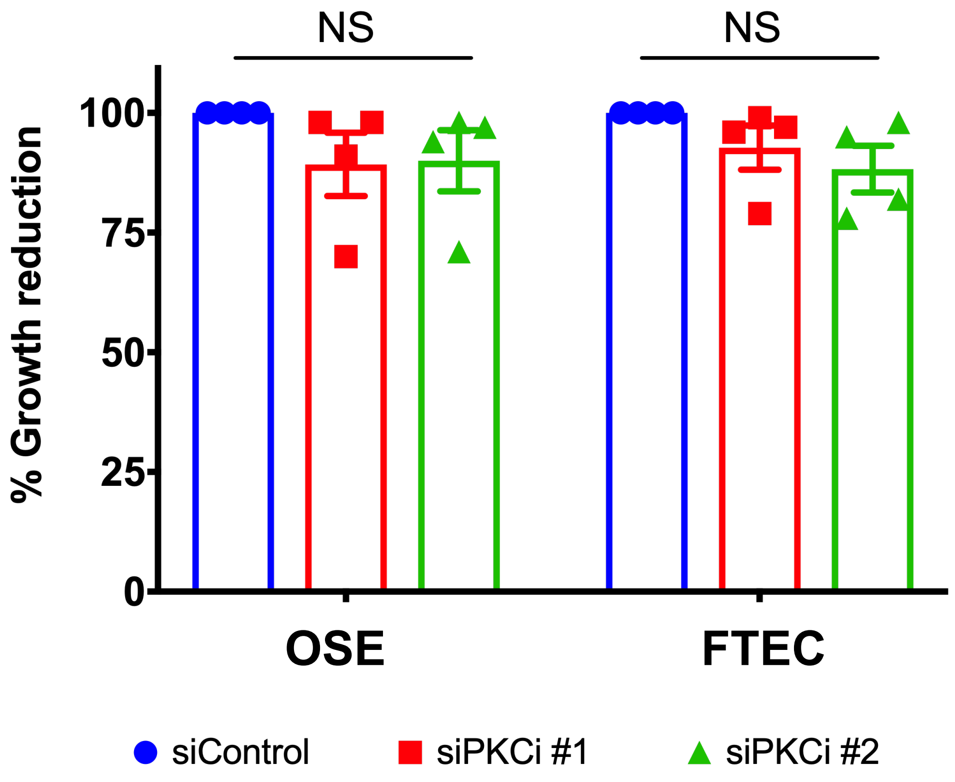

Figure S3. Sensitivity of silencing PKCι to cell growth in OSE and FTECs. Cell growth analysis was performed on OSE and FTECs. The percent growth was expressed as 84-h values relative to 12-h values. The value of PKCι siRNA-treated cells were compared relative to Control siRNA-treated ones, which were normalized to 100%. Data are means ± SD. n = 4. Two-way ANOVA was used to analyze and NS indicates no statistical significance.

Supplementary Data Figure S4

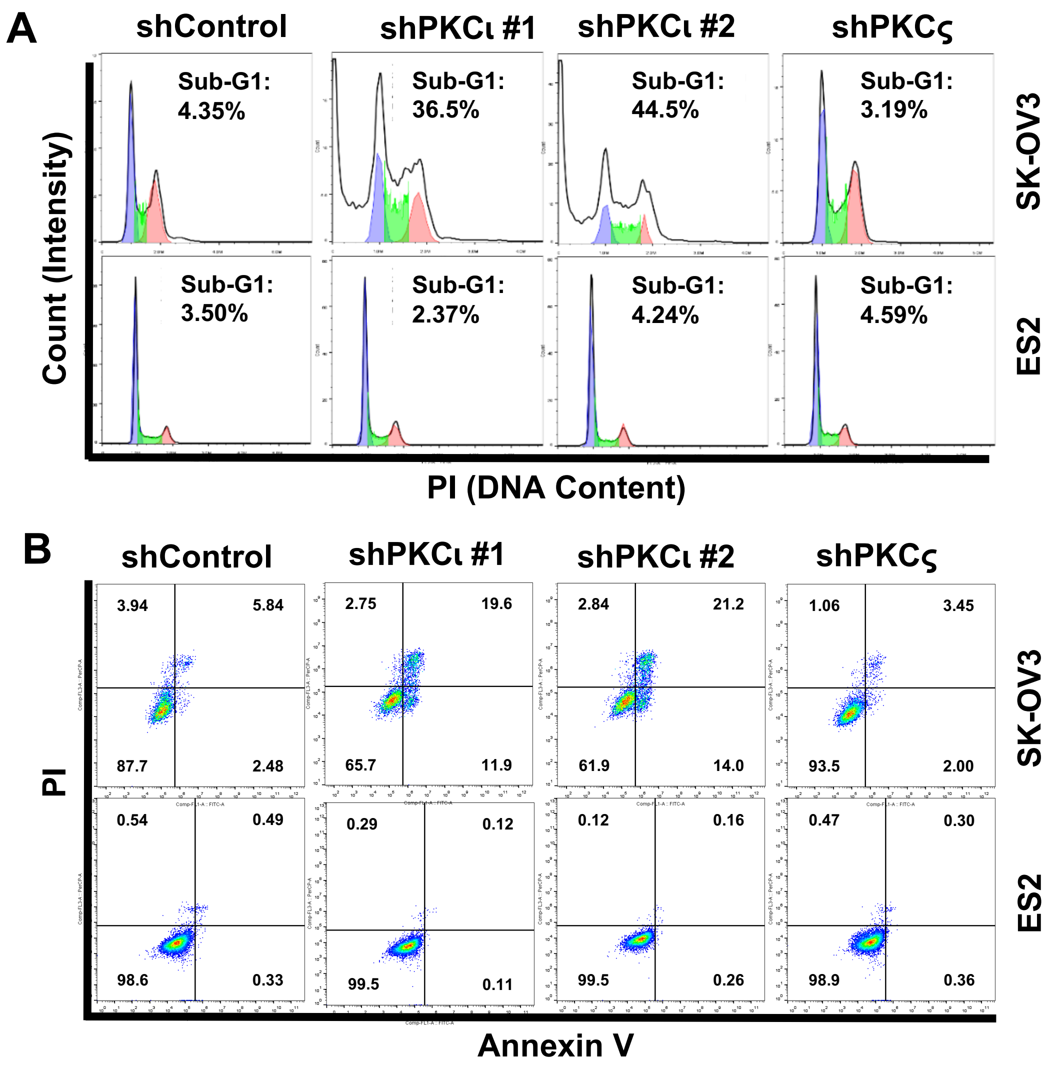

Figure S4. Knockdown of PKCι induces apoptosis in *PRKCI*-amplified ovarian cancer cells. ***A.*** Ovarian cancer cells were infected with lentiviral vector containing Scramble, PKCι or PKCς shRNA. Cells were detached 4 days of post-infection and then subjected to flow cytometry to analyze cell cycle progression. The horizontal axis is represented by PI (DNA content) and the vertical axis is represented by Count (Intensity). Results are representative of three independent experiments. ***B.*** Ovarian cancer cells were infected with lentiviral vector containing Scramble, PKCι or PKCς shRNA. Cells were detached 4 days of post-infection and subjected to Annexin V/PI-based flow cytometry. The horizontal axis is represented by Annexin V and the vertical axis is represented by PI. Cells in Quadrant 2 and 3 represent apoptotic cells. Results are representative of three independent experiments.

Supplementary Data Figure S5

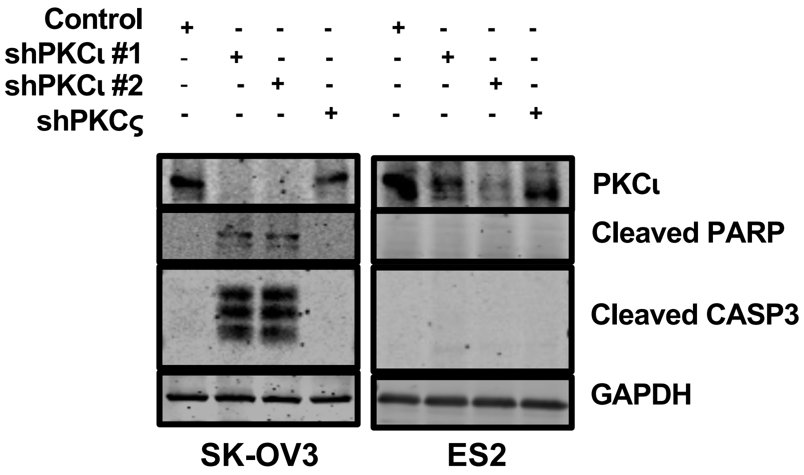

Figure S5. Knockdown of PKCι induces apoptosis in *PRKCI*-amplified ovarian cancer cells. Ovarian cancer cells were infected with lentiviral vector containing Scramble, PKCι or PKCς shRNA. After 4 days of infection, cells were lused and cell lysates were subjected to Western blotting to detect PKCι, cleaved PARP, cleaved CASP3 and GAPDH with the respective antibodies.

Supplementary Data Figure S6

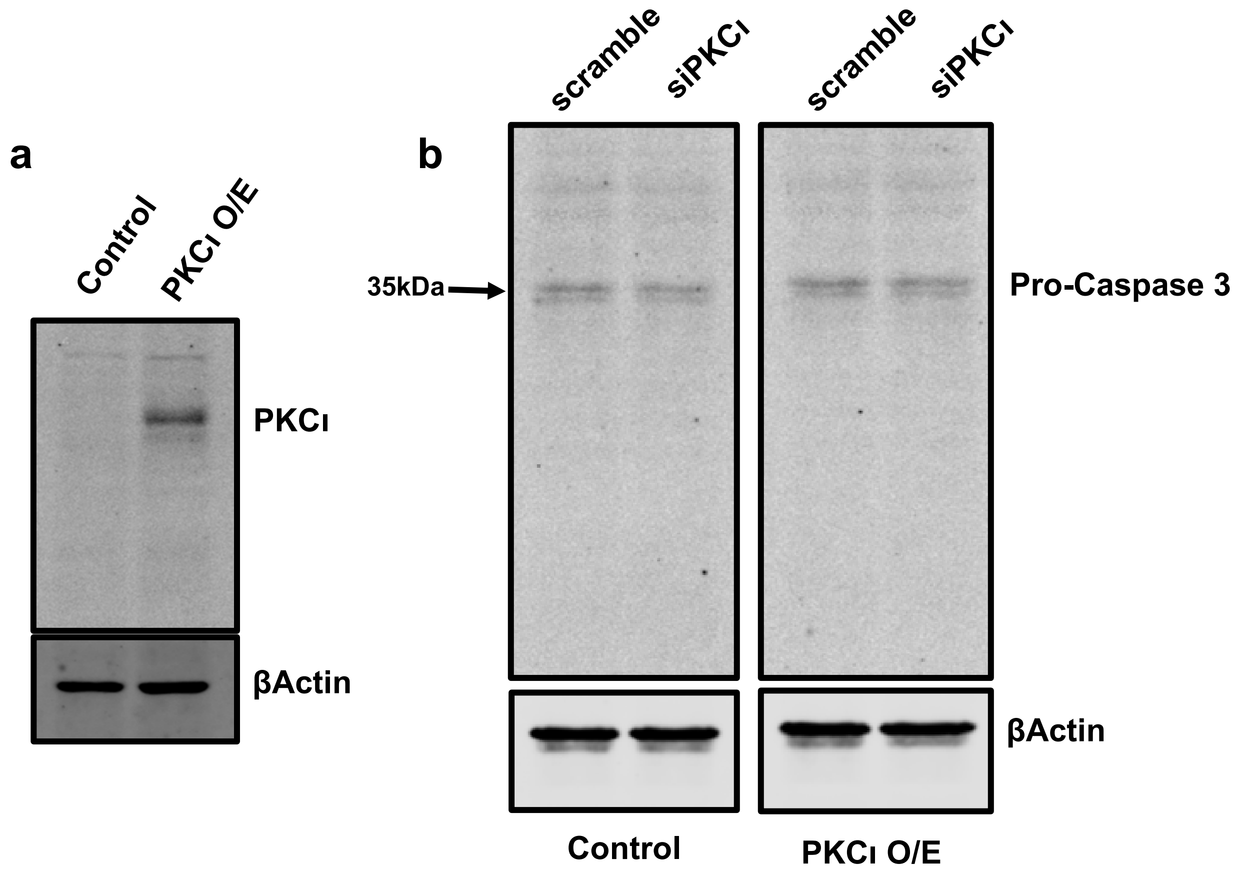

Figure S6. Increasing *PRKCI* copies is insufficient to sensitive ovarian cancer cells to PKCι knockdown. ***A*.** PKCι-containing vector or empty vector was lentivirally introduced into OVCA429 cells. Cells were harvested and cell lysates were subjected to Western blot to detect PKCι. ***B***. Empty vector (control) and PKCι-transduced cells (PKCι O/E) were transfected with scramble or PKCι siRNA pool (siPKCι) for 4 days followed by Western blotting to detect CASP3.

Supplementary Data Figure S7

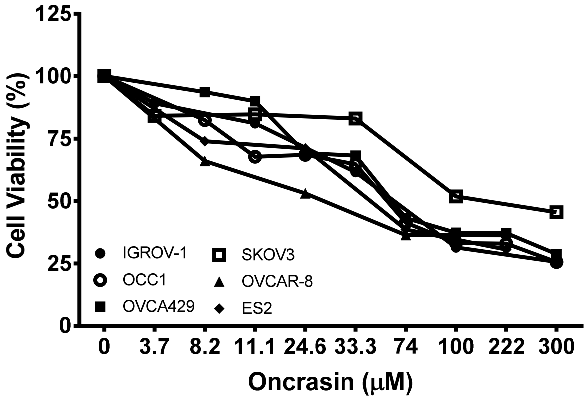

Cell Line	Oncrasin IC_{50} (μM)
OVCAR-8	33.39
ES2	45.53
IGROV1	54.45
OCC1	62.95
OVCA429	73.72
SKOV3	182.83

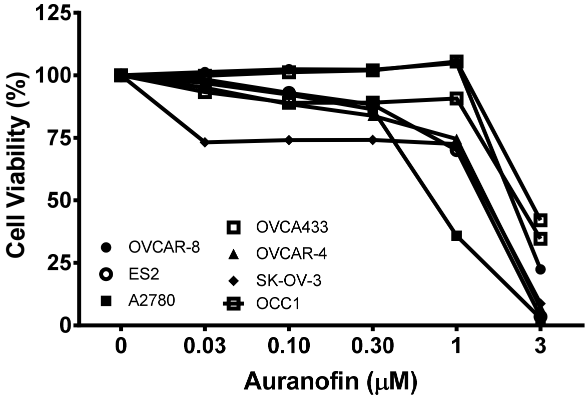

Cell Line	ANF IC_{50} (μM)
A2780	0.78
ES2	1.31
OVCAR-4	1.50
SK-OV-3	1.75
OVCAR-8	2.62
OCC1	2.62
OVCA433	2.88

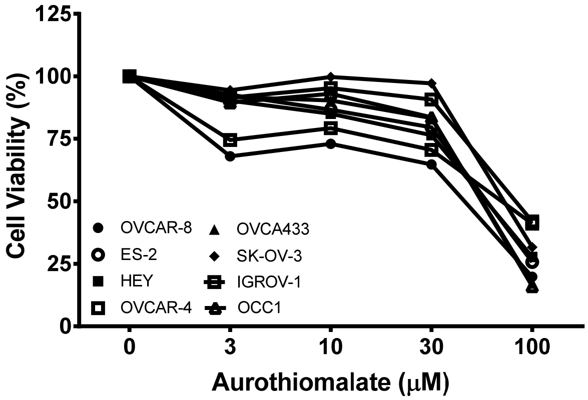

Cell Line	ATM IC_{50} (μM)
OVCAR-8	40.23
OCC1	59.12
OVCA433	59.64
HEY	63.39
ES-2	63.51
SK-OV-3	84.92
IGROV-1	88.35
OVCAR-4	91.01

Figure S7. Inhibitory effect of Oncrasin, ANF and ATM in ovarian cancer cell lines. Cells were treated with each individual inhibitor at various concentrations. The cell viability was expressed as 84-hour values relative to 12 hour values for each cell line. Data are means ± SD. n = 3. IC50 values were calculated using GraphPad software.

Supplementary Data Figure S8

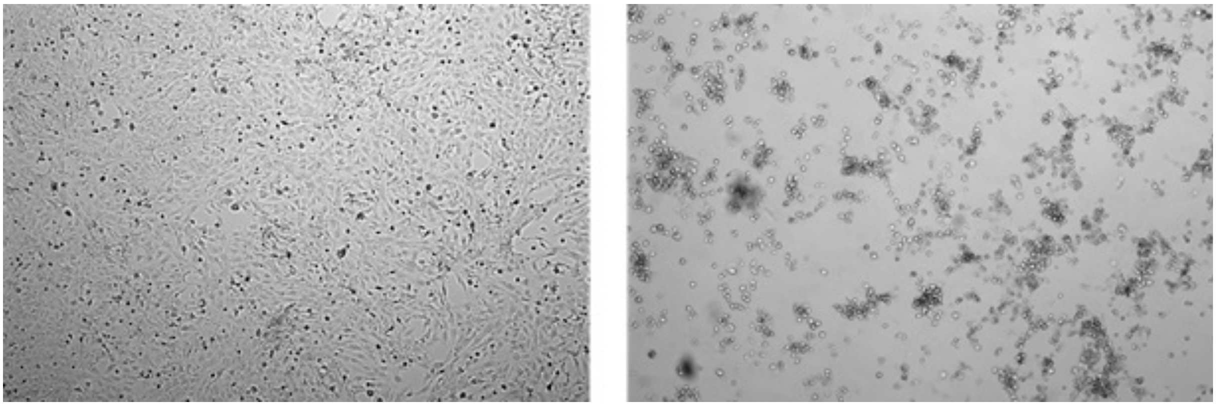

Vehicle (DMSO) ANF

Figure S8. ANF is cytotoxic to FIECs. FTECs were treated with Vehicle (DMSO) or ANF at 1μM and images using the microscope at 5X were taken 2 days later.

Supplementary Data Figure S9

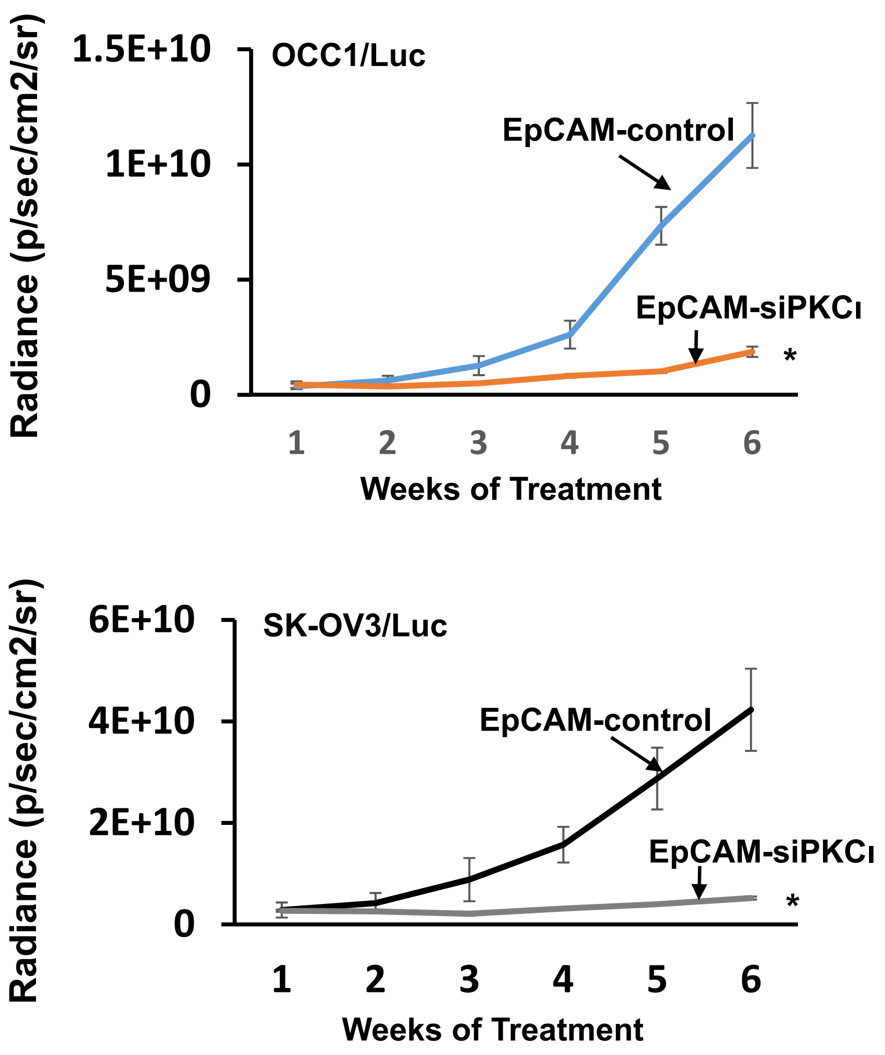

Figure S9. Effect of aptamer treatment on intraperitoneal xenograft development. Once tumor was detected in the mice, they were divided into two groups (5 per group) and received either EpCAM-control or EpCAM-siPKCι aptamer thrice a week intraperitoneally (200nmole/mouse). Tumor outgrowth was monitored weekly using the Xenogen IVIS-200 *In Vivo* bioluminescence imaging system. Error bars represent standard errors. * indicates $P < 0.005$ vs EpCAM control.

Supplementary Data Figure S10

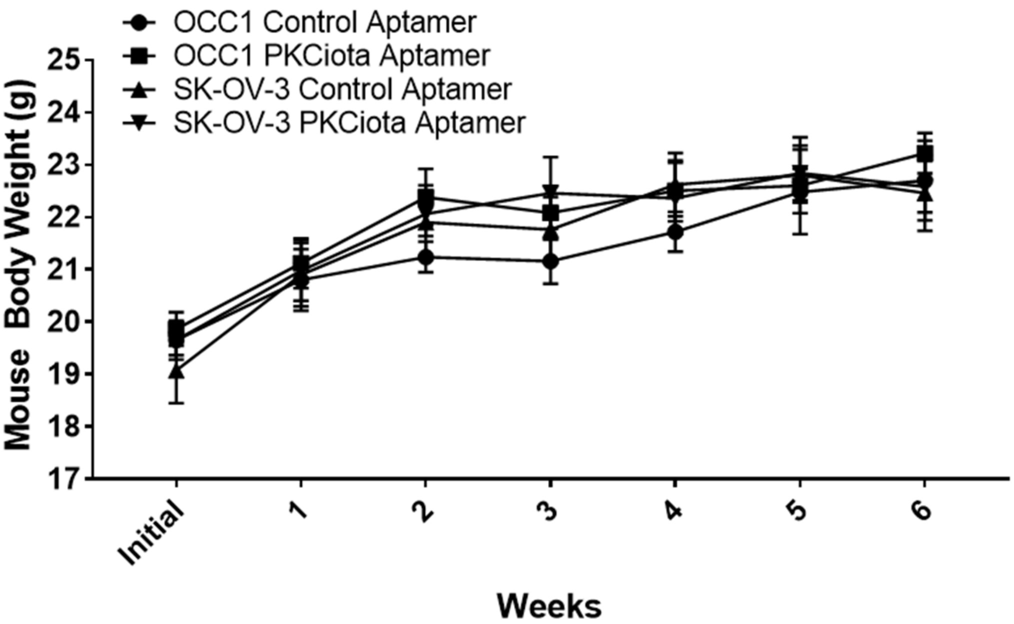

Figure S10. Effect of aptamer treatment on body weight of mice. Weights of the mice were measured every week of the experiment and no significant differences were noticed when control and EpCAM-siPKCι aptamer treated mice were compared. Error bars represent standard errors.

Supplementary Data Figure S11

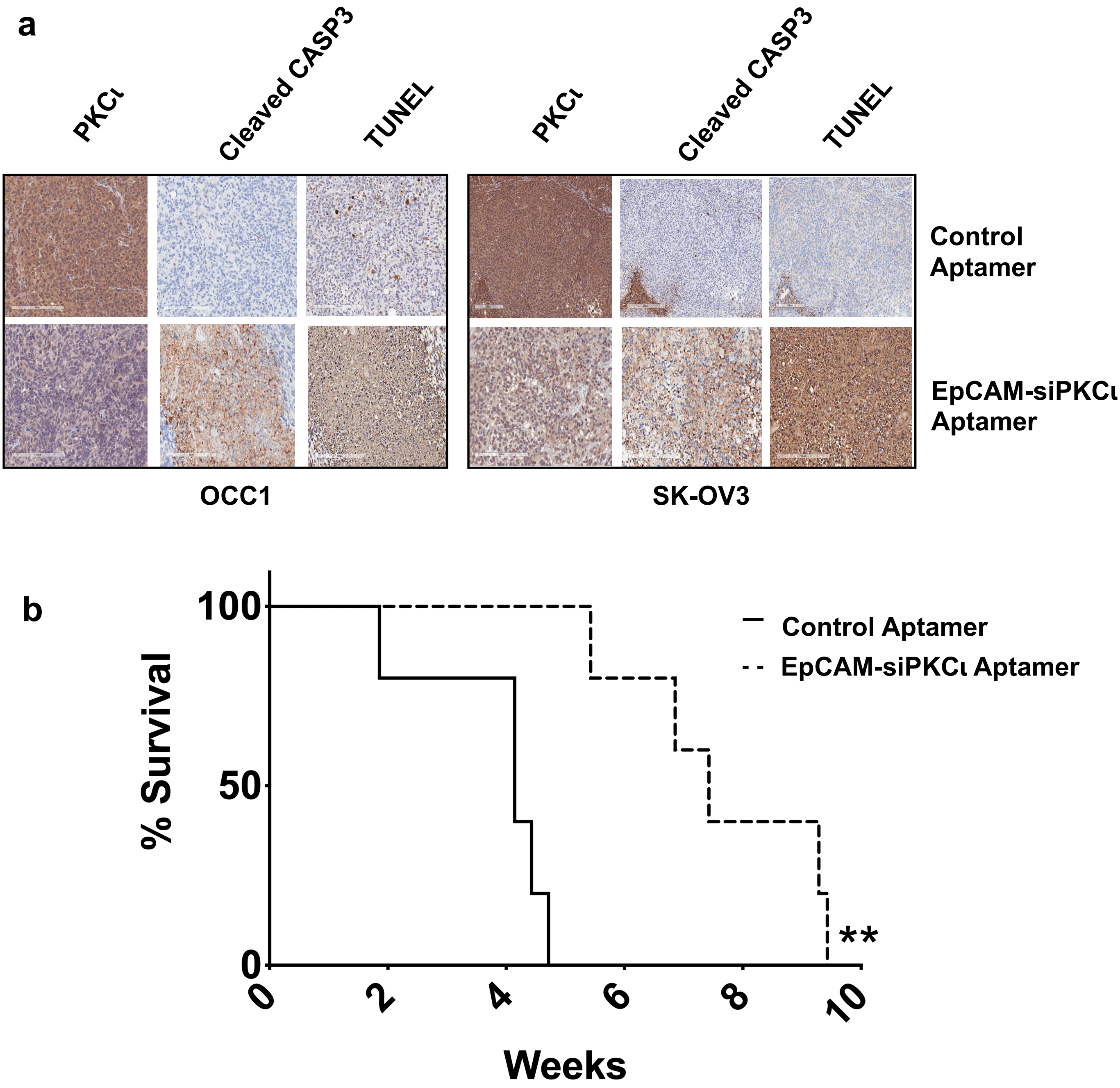

Figure S11. EpCAM aptamer-delivered PKCι siRNA prolongs lifespan of tumor-bearing mice. ***a***. Representative pictures of IHC staining on PKCι, cleaved CASP3 and TUNEL in tumor tissues derived from OCC1 and SK-OV3 cells. Scale bars, 50 μm. ***b***. Kaplan-Meier analysis of animal endpoint survival following treatment with control or EpCAM-siPKCι aptamer in athymic nude mice injected with luciferase-expressing OCC1 cells. n = 5. ** indicates $P < 0.01$ vs Control Aptamer.

Supplementary Data Table S1

Table S1. Primary and Secondary Antibodies used

Primary Antibody Name	Isotype (IgG)	Company and Catalog Number	MW (kDa)	Dilution Ratio
Cleaved Caspase 3 (Asp175) (5A1E)	Rabbit	Cell Signaling 9664	17, 19	(1:1000)
GAPDH (D4C6R)	Mouse	Cell Signaling 97166	37	(1:2000)
EpCAM/CD326	Mouse	Cell Signaling 2929	40	(1:1000)
β-Actin (8H10D10)	Mouse	Cell Signaling 3700	45	(1:2000)
Anti-PRKCI	Rabbit	Millipore Sigma ABC472	66	(1:500)
PKC zeta (C24E6)	Rabbit	Cell Signaling 9368	78	(1:1000)
Cleaved PARP (Asp214) (D64E10)	Rabbit	Cell Signaling 5625	89	(1:500)

Secondary Antibody Name	Isotype (IgG)	Company	Catalog #	Dilution Ratio
IRDye 680RD Secondary Antibody	Goat anti-Mouse	LICOR	926-68070	1:20,000
IRDye 800CW Secondary Antibody	Goat anti-Rabbit	LICOR	926-32211	1:20,000
